# Frequency of Going Outdoors and Risk of Poor Oral Health Among Older Japanese Adults: A Longitudinal Cohort From the Japan Gerontological Evaluation Study

**DOI:** 10.2188/jea.JE20220221

**Published:** 2024-02-05

**Authors:** Keiko Ishimura, Ryoto Sakaniwa, Kokoro Shirai, Jun Aida, Kenji Takeuchi, Katsunori Kondo, Hiroyasu Iso

**Affiliations:** 1 Department of Social Medicine, Osaka University Graduate School of Medicine, Osaka, Japan; 2 Department of Oral Health Promotion, Tokyo Medical and Dental University Graduate School of Medical and Dental Science, Tokyo, Japan; 3 Department of International and Community Oral Health, Tohoku University Graduate School of Dentistry, Sendai, Japan; 4 Division of Statistics and Data Science, Liaison Center for Innovative Dentistry, Tohoku University Graduate School of Dentistry, Sendai, Japan; 5 Department of Preventive Medicine, Nagoya University Graduate School of Medicine, Nagoya, Japan; 6 Department of Social Preventive Medical Sciences, Center for Preventive Medical Sciences, Chiba University, Chiba, Japan; 7 Department of Gerontological Evaluation, Center for Gerontology and Social Science, National Center for Geriatrics and Gerontology, Aichi, Japan; 8 Institute for Global Health Policy Research, Bureau of International Health Cooperation, National Center for Global Healthcare Center Medicine, Tokyo, Japan

**Keywords:** going outdoors, oral health, older adult, mediation effect, longitudinal study

## Abstract

**Background:**

The association between the frequency of going outdoors and the risk of poor oral health has been reported in several studies; however, the findings have been inconclusive.

**Methods:**

We conducted a 3-year longitudinal study of 19,972 Japanese adults aged ≥65 years who reported no poor oral condition at baseline in 2013. The respondents rated their frequency of going outdoors in three categories (≤1, 2–3, or ≥4 times/week), and the oral conditions reported in 2016 included tooth loss, chewing difficulty, swallowing difficulty, dry mouth, and composite outcomes. The associations between the frequency of going outdoors and the risk of poor oral health were examined as relative risk ratios (RRs) and 95% confidence intervals (CIs) using multivariable Poisson regression, while mediation analysis was performed to investigate indirect effects.

**Results:**

During the follow-up, 32.5% of participants developed poor oral health. In the mediation analysis, indirect effects were observed through low instrumental activities of daily living, depressive symptoms, little social network diversity, and underweight. Compared to going outdoors ≥4 times/week, the multivariable RRs of composite poor oral health conditions were 1.12 (95% CI, 1.05–1.20) for 2–3 times/week and 1.22 (95% CI, 1.07–1.39) for ≤1 time/week (*P*-trend < 0.001). Similar associations were observed for tooth loss, chewing difficulty, and swallowing difficulty; the corresponding RRs were 1.07 (95% CI, 0.97–1.19) and 1.36 (95% CI, 1.13–1.64) (*P*-trend = 0.002), 1.18 (95% CI, 1.06–1.32) and 1.30 (95% CI, 1.05–1.60) (*P*-trend < 0.001), and 1.15 (95% CI, 1.01–1.31) and 1.38 (95% CI, 1.08–1.77) (*P*-trend = 0.002), respectively.

**Conclusion:**

The frequency of going outdoors was inversely associated with the risk of poor oral health through several modifiable risk factors in the older population.

## INTRODUCTION

Poor oral health conditions, such as having less than 20 teeth, and chewing and swallowing difficulties could cause poor nutritional status,^1^ leading to physical frailty^2^ and low health-related quality of life.^3^ Maintaining healthy oral conditions and treatment at an early stage are encouraged in older people.^4^

The association between poor oral health and the risk of homebound status (going outdoors less than once a week)^5^^,^^6^ and the opposite association^6^ have been reported previously by the Japan Gerontological Evaluation Study (JAGES). Homebound status or going outdoors less frequently is associated with reduced muscle mass,^7^ increased blood glucose levels,^8^ poor self-oral care^9^ and professional care,^10^ and reduced sunlight exposure,^11^^,^^12^ which are risk factors for poor oral health. However, no study has prospectively examined the association between frequency of going outdoors and the risk of poor oral health.

Therefore, this study examined this association and the indirect effects of other risk factors on poor oral health among 20,000 older Japanese adults in a community-based prospective cohort study. We hypothesized that going outdoors less frequently could predict the risk of subsequent poor oral health.

## METHODS

### Study sample

For this prospective cohort study, we used data from the JAGES, which has been described in detail elsewhere.^13^ We recruited 21,582 individuals, aged ≥65 years with no physical or cognitive disabilities and no poor oral health conditions at baseline in 2013, in 30 local areas in Japan. We excluded 830 participants who lacked information on their frequency of going outdoors and 780 who lacked oral assessments in 2016. Finally, for the main analysis, we included 19,972 individuals: 8,957 men and 11,015 women (eFigure 1).

To investigate whether the association between the frequency of going outdoors and the risk of poor oral health was affected by the frequency of going outdoors 3 years prior, we collected information on the frequency of going outdoors in 2010 for a subsample of 7,176 (in 16 areas) from 19,972 (in 30 areas). All participants were informed that participation in the study was voluntary and that completing and returning the self-administered questionnaire via mail would indicate their consent to participate. Ethics approval was obtained from the ethics committees of Nihon Fukushi University (No. 10-05) and Chiba University (No. 1777).

### Measurements

#### Dependent variable: oral health

We administered a self-report questionnaire to all participants in 2013 and 2016 regarding their oral health condition. Four oral health indicators were selected based on the criteria of oral hypofunction and frailty, according to the Japanese Society of Gerodontology.^14^ Tooth loss was defined as the presence of <20 remaining teeth. We asked “how many teeth do you currently have?” As an answer, subjects selected from ‘no remaining teeth,’ ‘1–4 remaining teeth,’ ‘5–9 remaining teeth,’ ‘10–19 remaining teeth,’ or ‘≥20 remaining teeth’. Chewing difficulty was defined as an answer of yes to “have you experienced more difficulty in chewing tough foods than 6 months ago?”. Swallowing difficulty was defined as an answer of yes to “have you ever experienced choking or coughing when drinking tea or soup?”. Dry mouth was defined as yes to “are you bothered by feelings of thirst or dry mouth?” For the primary assessment, we evaluated the risk of composite poor oral conditions as a binomial variable, with or without at least one of the above oral health conditions, and the risk of cumulative poor oral health on a multinomial scale.

#### Independent variable: frequency of going outdoors

At the baseline, we asked all participants “how often do you usually go outdoors?” with six responses: ≥4 times a week, 2–3 times a week, once a week, 1–3 times a month, several times a year, and never. For our analyses, we reclassified them into three categories: ≥4 times a week, 2–3 times a week, and ≤1 time a week. For changes in the frequency of going outdoors from 3 years before the baseline, we asked respondents to a frequency of ≤4 times/week, 2–3 times/week, or ≥1 time/week in 2010. Subsequently, we divided the participants into nine categories to combine the frequencies of going outdoors in 2010 and 2013.

### Mediators

We examined the mediating effects of being underweight, obesity, low instrumental activities of daily living (IADL), falls within 1 year, depressive symptoms, and little social network diversity, on the association between the frequency of going outdoors and poor oral health. Being underweight and obesity were defined by body mass index (BMI). We recorded being underweight as “20 < BMI < 25; coded 1,” “18.5 < BMI ≤ 20; coded 2,” and “BMI ≤18.5; coded 3.” Similarly, obesity was recorded as “20 < BMI < 25; coded 1,” “25 ≤ BMI < 30; coded 2,” and “BMI ≥30 kg/m^2^; coded 3.” We assessed IADL with subscales of the Tokyo Metropolitan Institute of Gerontology Index of Competence (0–5 points)^15^: “5 points; coded 1,” “4 points; coded 2,” and “0–3 points; coded 3.” Falls within 1 year were recorded as “Nothing; coded 1,” “One time; coded 2,” and “≥2 times; coded 3.” Also, we recorded the depressive symptoms (defined as Geriatric Depression Scale-15^16^): “0–4 points; coded 1,” “5–9 points; coded 2,” and “≥10 points; coded 3.” Further, we defined the number of types of friends and acquaintances as social network diversity^17^: “≥3 types; coded 1,” “2 types; coded 2,” and “0–1 types; coded 3.” The types of friends and acquaintances were classified as neighbors, childhood friends, school friends, colleagues, those sharing the same interests or leisure activities, and those involved in the same volunteer activities.

### Confounders

The analysis was adjusted for the following confounders: age (65–74 or ≥75 years), sex, smoking status (current, past, or never smoking), years of education (≤9, 10–12, or ≥13 years), and annual household income (low: ≤19,999 USD, middle: 20,000–39,999 USD, high: ≥49,999 USD; 1 USD ≒ 100 JPY). We categorized the missing data for these mediators and covariates as “missing data.”

### Statistical analysis

We used the χ^2^ test for categorical variables and one-way ANOVA for continuous variables to test the differences in the frequency of going outdoors at baseline. The association between the frequency of going outdoors and the risks of composite poor oral health conditions, tooth loss, chewing difficulty, swallowing difficulty, and dry month were reported as relative risk ratios (RRs) and 95% confidence intervals (CIs) using multivariable-adjusted Poisson regression.^18^ We calculated the risk of poor oral health in the age- and sex-adjusted and multivariable adjusted models.

We performed mediation analysis^19^ to investigate the percentage of indirect effects via mediating factors between the frequency of going outdoors and the risk of cumulative poor oral health conditions, using multiple regression to examine the association.

Finally, we performed a subsample analysis of the associations between changes in the frequency of going outdoors during the past 3 years before baseline and the risk of composite poor oral health conditions, with Poisson regression among 7,176 of 19,972 individuals. Statistical significance was set at *P*-value < 0.05, and all analyses were performed using SAS version 9.4 (SAS Institute Inc., Cary, NC, USA).

## RESULTS

The mean age of participants in this study was 71.5 years. Over 3 years of follow-up, 6,482 subjects (32.5%) newly developed poor oral health (tooth loss, *n* = 2,773; chewing difficulty, *n* = 2,372; swallowing difficulty, *n* = 1,679; dry mouth, *n* = 1,901).

Table 1 shows the baseline characteristics according to the frequency of going outdoors: lower frequency of going outdoors was associated with female sex, older age, smoking, low education, low annual household income, underweight (BMI ≤18.5 kg/m^2^), obesity (BMI ≥30 kg/m^2^), low IADL, fall within 1 year, depressive symptoms, and little social network diversity.

**Table 1.** The baseline characteristics according to the frequency of going outdoors

	Frequency of going outdoors			

	≤1 time/week	2–3 times/week	≥4 times/week	*P*-trend
Number at risk, *n*	586	2,768	16,618	
Male, %	47.1	39.2	45.7	<0.001
Mean age, years	72.9	72.2	71.3	<0.001
65–74, %	65.4	69.5	77.2	
≥75, %	34.6	30.5	22.6	
Smoking status, %				<0.001
Current smoking	7.7	6.9	6.7	
Past smoking	13.8	12.0	15.0	
Never smoking	78.0	80.7	77.9	
Years of education, %				<0.001
≤9 years	38.1	33.6	30.8	
10–12 years	38.7	41.1	41.9	
≥13 years	21.2	23.7	26.4	
Annual inequivalent income, %				<0.001
Low (≤19,999 USD)	44.2	42.9	36.4	
Middle (20,000–39,999 USD)	32.9	36.2	40.6	
High (≥40,000 USD)	8.2	8.7	12.4	
Mean body mass index, kg/m^2^	23.1	22.8	22.9	<0.001
Underweight (BMI ≤18.5), %	7.3	7.0	5.2	<0.001
Underweight tendency (18.5 < BMI ≤ 20), %	64.9	69.7	72.3	
Obesity tendency (25 ≤ BMI < 30), %	18.9	18.9	18.9	<0.001
Obesity (BMI ≥30), %	3.5	1.9	1.8	
IADL, %				<0.001
Little low (4 points)	14.9	9.7	7.5	
Low (0–3 points)	10.8	2.9	1.6	
Fall within 1 year, %				<0.001
1 time	13.3	13.5	12.8	
≥2 times	3.8	1.7	1.8	
Geriatric Depression Scale-15				<0.001
Minor depressive symptoms (5–9 points), %	64.7	63.6	54.7	
Major depressive symptoms (≥10 points), %	8.0	5.0	2.5	
Social network diversity, %				<0.001
0–1	59.0	46.9	34.8	
2	24.9	33.5	34.3	
≥3	9.4	17.0	28.7	

BMI, body mass index. IADL, instrumental activities for daily living. 1 USD ≒ 100 JPY.

IADL was measured using the subscales of the Tokyo Metropolitan Institute of Gerontology Index of Competence (5 points). Social network diversity was defined as the number of types of friends and acquaintances.

Table 2 presents the results of the mediation analysis. The significant indirect effects of the modifiable risk factors on the risk of poor oral health were as follows: low IADL, 12.9% (95% CI, 7.9–21.0%); depressive symptoms 12.5% (95% CI, 6.4–23.1%); little social network diversity, 9.0% (95% CI, 4.0–17.0%); and underweight, 2.3% (95% CI, 0.2–1.2%). In contrast, no significant indirect effects were found for fall within a year (1.3%; 95% CI, −0.6% to 1.6%) and obesity (0.1%; 95% CI, −0.1% to 0.5%).

**Table 2.** Percentage of indirect effects and 95% interval confidences (95% CIs) via mediating other risk factors between frequency of going outdoors and the risk of accumulative poor oral health conditions

Subjects	Indirect effect (95% CI), %
Underweight (≤18.5 kg/m^2^)	2.3 (0.2–1.2)
Obesity (≥30 kg/m^2^)	0.1 (−0.1 to 0.5)
Low IADL (<5 points)	12.9 (7.9–21.0)
Fall within 1 year (≥1 time)	1.3 (−0.6 to 1.6)
Depressive symptoms (≥5 points)	12.5 (6.4–23.1)
Little social network diversity (≤2 types)	9.0 (4.0–17.0)

IADL, instrumental activities for daily living.

All indirect effects were adjusted for age, sex, smoking status, years of education, and annual household income.

The definitions for being underweight and obese were defined by body mass indexes. Low IADL was defined using the subscales of the Tokyo Metropolitan Institute of Gerontology Index of Competence. Depressive symptoms were defined using the Geriatric Depression Scale-15. Little social network diversity was defined by the number of types of friends and acquaintances.

Table 3 shows the relative risks of poor oral health according to the frequency of going outdoors. In the age- and sex-adjusted model, we found inverse associations between the frequency of going outdoors and the risk of each poor oral health condition. When adjusted for other confounders, the association remained significant with composite poor oral health conditions, tooth loss, chewing difficulty, and swallowing difficulty. The multivariable RRs of composite poor oral health conditions were 1.12 (95% CI, 1.05–1.20) for 2–3 times/week and 1.22 (95% CI, 1.07–1.39) for ≤1 time/week (*P*-trend < 0.001) compared to a frequency of ≥4 times/week. The corresponding RRs of tooth loss, chewing difficulty, and swallowing difficulty were 1.07 (95% CI, 0.97–1.19) and 1.36 (95% CI, 1.13–1.64) (*P*-trend = 0.002), 1.18 (95% CI, 1.06–1.32) and 1.30 (95% CI, 1.05–1.60) (*P*-trend < 0.001); and 1.15 (95% CI, 1.01–1.31) and 1.38 (95% CI, 1.08–1.77) (*P*-trend = 0.002). Similar but weaker associations were observed with dry mouth.

**Table 3.** Multivariable relative risk ratios for the risk of composite poor oral health conditions, tooth loss, chewing difficulty, swallowing difficulty, and dry mouth according to the frequency of going outdoors

	Frequency of going outdoors			

	≤1 time/week	2–3 times/week	≥4 times/week	*P*-trend
Number at risk	586	2,768	16,618	
**Composite poor oral health conditions**				
Number of incidence (%)	235 (40.1%)	1,006 (36.3%)	5,241 (31.5%)	
Age- and sex-adjusted RR (95% CI)	1.25 (1.09–1.42)	1.14 (1.06–1.22)	Reference	<0.001
Multivariable RR (95% CI)	1.22 (1.07–1.39)	1.12 (1.05–1.20)	Reference	<0.001
**Tooth loss**				
Number of incidence (%)	116 (19.8%)	416 (15.0%)	2,241 (13.5%)	
Age- and sex-adjusted RR (95% CI)	1.42 (1.18–1.71)	1.10 (1.00–1.22)	Reference	<0.001
Multivariable RR (95% CI)	1.36 (1.13–1.64)	1.07 (0.97–1.19)	Reference	0.002
**Chewing difficulty**				
Number of incidence (%)	93 (15.9%)	385 (13.9%)	1,894 (10.9%)	
Age- and sex-adjusted RR (95% CI)	1.36 (1.10–1.67)	1.21 (1.08–1.35)	Reference	<0.001
Multivariable RR (95% CI)	1.30 (1.05–1.60)	1.18 (1.06–1.32)	Reference	<0.001
**Swallowing difficulty**				
Number of incidence (%)	67 (11.4%)	262 (9.5%)	1,350 (8.1%)	
Age- and sex-adjusted RR (95% CI)	1.40 (1.09–1.78)	1.16 (1.01–1.32)	Reference	<0.001
Multivariable RR (95% CI)	1.38 (1.08–1.77)	1.15 (1.01–1.31)	Reference	0.002
**Dry mouth**				
Number of incidence (%)	65 (11.1%)	343 (12.4%)	1,493 (9.0%)	
Age- and sex-adjusted RR (95% CI)	1.20 (0.93–1.53)	1.34 (1.19–1.51)	Reference	<0.001
Multivariable RR (95% CI)	1.18 (0.92–1.52)	1.33 (1.18–1.50)	Reference	<0.001

CI, confidence interval; RR, risk ratio.

All RRs were estimated using Poisson regression. Multivariate RRs were adjusted for age, sex, smoking status, years of education, and annual household income.

Composite poor oral health conditions include tooth loss, chewing difficulty, swallowing difficulty, and/or dry mouth.

Table 4 shows results of the associations between the frequency of going outdoors and the risk of accumulative poor oral health. The multivariable RRs of any single poor oral health condition were 1.06 (95% CI 0.98–1.15) and 1.10 (95% CI, 0.93–1.29) (*P*-trend = 0.083). The corresponding multivariable RRs of 2–4 poor oral health conditions were 1.53 (95% CI, 1.22–1.91) and 1.29 (95% CI, 1.14–1.46) (*P*-trend < 0.001).

**Table 4.** Multivariable relative risk ratios for the risk of accumulative poor oral health conditions according to the frequency of going outdoors

Number of accumulative poor oral health conditions	Frequency of going outdoors			

	≤1 time/week	2–3 times/week	≥4 times/week	*P*-trend
Number at risk	586	2,768	16,618	
**One**				
Number of incidence (%)	154 (26.3%)	693 (25.0%)	3,850 (23.2%)	
Age- and sex-adjusted RR (95% CI)	1.12 (0.95–1.31)	1.07 (0.99–1.16)	Reference	0.041
Multivariable RR (95% CI)	1.10 (0.93–1.29)	1.06 (0.98–1.15)	Reference	0.083
**Two-Four**				
Number of incidence (%)	81 (13.8%)	313 (11.3%)	1,391 (8.4%)	
Age- and sex-adjusted RR (95% CI)	1.60 (1.27–2.00)	1.33 (1.17–1.50)	Reference	<0.001
Multivariable RR (95% CI)	1.53 (1.22–1.91)	1.29 (1.14–1.46)	Reference	<0.001

CI, confidence interval; RR, risk ratio.

All RRs were estimated using Poisson regression. Multivariate RRs were adjusted for age, sex, smoking status, years of education, and annual household income.

Accumulative poor oral conditions were defined as the total number of poor oral conditions, including tooth loss, chewing difficulty, swallowing difficulty, and dry mouth.

**Figure 1.**
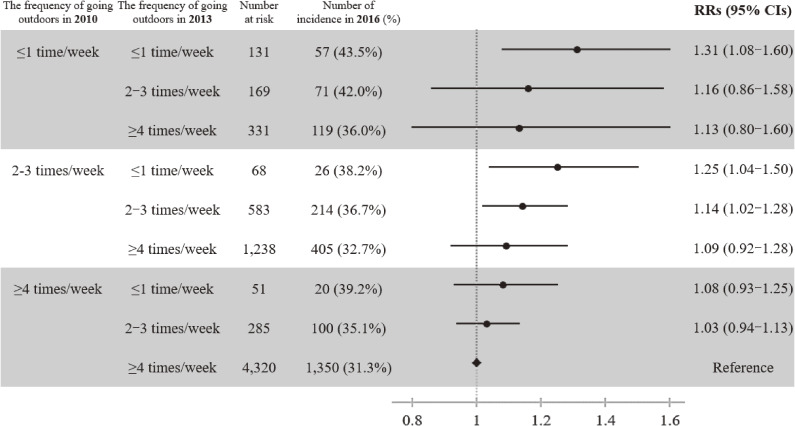
The subsamples analysis for relative risk ratios for composite poor oral health conditions according to change in the frequency of going outdoors between 2010 and 2013, compared to the frequency of going outdoors ≥4 times/week in both years (*n* = 7,176/19,972). All RRs were estimated with Poisson regression. CI, confidence interval; RR, risk ratio.

Figure 1 illustrates the multivariable RRs of composite poor oral health conditions according to the frequency of going outdoors in 2013 stratified by that in 2010. In this subsample analysis, the association between the lower frequency of going outdoors and the risk of poor oral health was more pronounced among participants who went outdoors ≤1 time/week in 2010.

## DISCUSSION

This study found that going outdoors less than once a week is associated with an increased risk of poor oral health. To our knowledge, this is the first study to find an association between the frequency of going outdoors and the risk of poor oral health among older adults using a large set of longitudinal cohort data. Our results extend the findings of a previous longitudinal study, in which the frequency of going outdoors was associated with poor oral health^6^ but the composite outcome was not examined. In addition, being homebound was associated with a prevalence of chewing difficulty (prevalence ratio [PR] 1.17; 95% CI, 1.05–1.30), but not with the prevalence of the number of teeth <20, choking experience, or dry mouth (PR 1.00; 95% CI, 0.95–1.04; PR 1.06; 95% CI, 0.92–1.22; and PR 1.02; 95% CI, 0.88–1.18, respectively).^6^

The direct mechanisms for the observed associations may be influenced by several factors. A low frequency of going outdoors could 1) reduce muscle mass,^7^ increase fatness,^20^ and increase blood glucose levels,^8^ which may decrease resistance to plaque bacteria, and increase the risk of periodontal disease^21^; 2) be associated with poor self-oral care,^9^ and reduce visits to a dentist^10^; and 3) reduce vitamin D production due to limited sunlight exposure,^11^^,^^12^ which may increase the risk of periodontal disease and tooth loss^22^^,^^23^ due to reduced calcium absorption and bone metabolism.

In our study, we found that the association between the frequency of going outdoors and the risk of poor oral health was mediated by modifiable risk factors, such as low IADL, depressive symptoms, little social network diversity, and being underweight (2–13% for each). These mediating mechanisms require further investigation. Low IADL and underweight are associated with sarcopenia, which leads to the reduction of muscle fibers in the jaw-opening force and swallowing muscle, causing difficulty in chewing and/or swallowing.^24^ Recently, several cross-sectional studies have shown that trunk^25^ and quadriceps^26^ muscle mass estimated using computed tomography imaging and ultrasound correlated with dysphagia in older inpatients. The mechanism is considered that the loss of whole-body muscle mass and secondary sarcopenia induced by less physical activity and malnutrition were associated with tongue atrophy^27^ and thinning of the pharyngeal wall,^28^ leading to dysphagia.

A history of depression was associated with a risk of tooth loss (odds ratio 1.31; 95% CI, 1.24–1.37).^29^ People with depressive symptoms are likely to have unhealthy lifestyle behaviors, including heavy alcohol consumption, high psychological stress, infrequent tooth brushing, and few dental visits, which are associated with periodontal disease^30^^,^^31^ and tooth loss.^32^ The diversity of social networks is associated with a larger number of remaining teeth,^17^ possibly by reducing psychological stress.^33^ Psychological stress responses may activate the hypothalamus-pituitary-adrenal axis to release cortisol, thereby enhancing inflammation in the periodontal tissues and increasing the risk of periodontal disease.^34^ A systematic review of eight case-control and 18 cross-sectional studies showed a positive association between stress-related biomarkers, such as cortisol and inflammatory interleukins, and the prevalence of periodontal disease.

### Strengths and limitations

The primary strength of our study is that we followed a large prospective cohort of nearly 20,000 participants from 30 municipalities in Japan, using multiple oral health outcomes and composite assessment and mediation analyses. However, this study has two limitations. First, the oral health assessments were self-reported; however, the clinical assessment for the number of teeth correlated strongly with self-reported oral health among both Japanese men (*ρ* = 0.78–0.82) and women (*ρ* = 0.77–0.91) estimated using Spearman’s rank correlation coefficient.^35^ Second, some of our results could be due to reverse causation because poor oral health was associated with future risk of homebound.^5^^,^^6^

### Conclusion

The results of this prospective study of 20,000 Japanese individuals aged ≥65 years found that a lower frequency of going outdoors was prospectively associated with a risk of poor oral health conditions. Additionally, these associations were mediated by modifiable risk factors, such as a low level of IADL, depressive symptoms, little social network diversity, and underweight.

## SUPPLEMENTARY MATERIAL

eFigure 1. The flowchart in the current study analyses
