# Supplementary figures and images for: Frequency of Going Outdoors and Risk of Poor Oral Health Among Older Japanese Adults: A Longitudinal Cohort From the Japan Gerontological Evaluation Study
Source: J Epidemiol. 2024 Feb 5;34(2):63–9. doi: 10.2188/jea.JE20220221 (PMC10751189; doi:10.2188/jea.JE20220221)

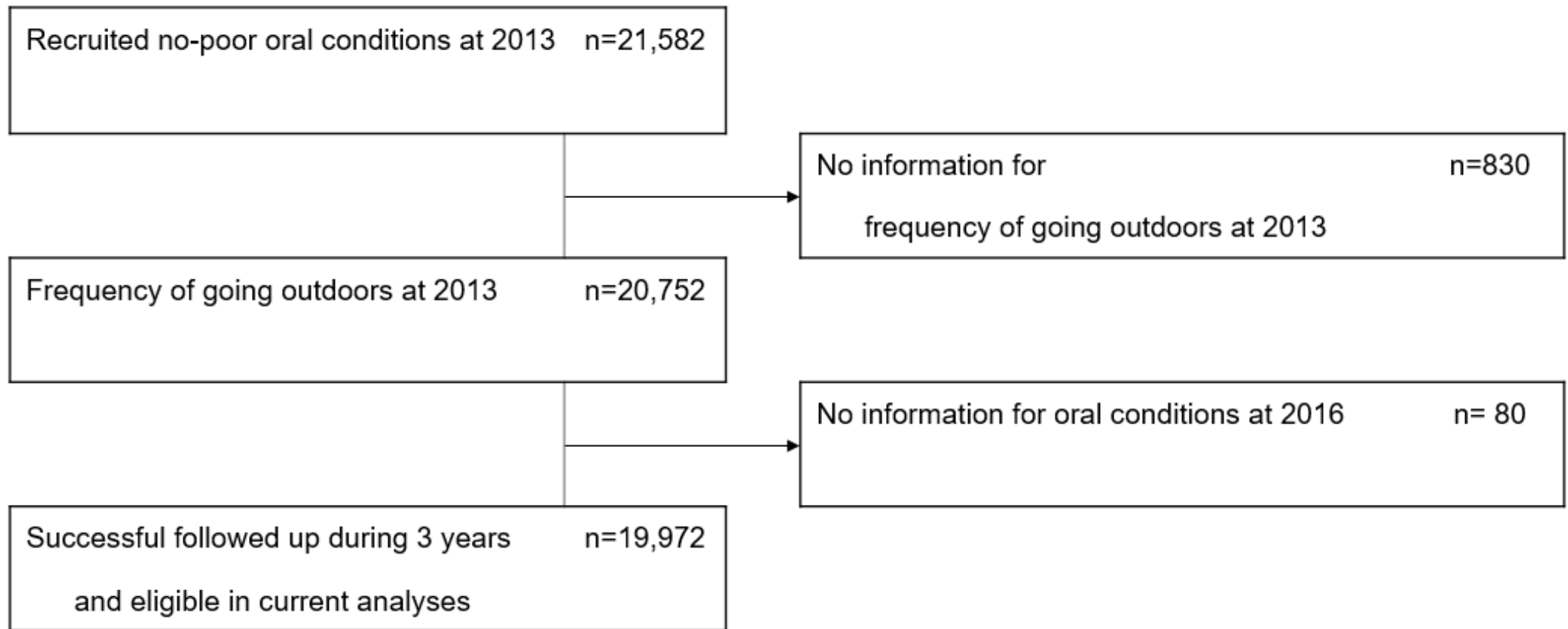

**eFigure 1.** The flowchart in the current study analyses

Supplement: Supplementary file 1 [file je-34-063-s001.pdf]
